# *Trypanosoma vivax* Adhesion to Red Blood Cells in Experimentally Infected Sheep

**DOI:** 10.1155/2016/4503214

**Published:** 2016-05-16

**Authors:** Alpidio A. Boada-Sucre, Marcello Salvatore Rossi Spadafora, Lucinda M. Tavares-Marques, Héctor J. Finol, Armando Reyna-Bello

**Affiliations:** ^1^Laboratorio de Microscopía Electrónica, Centro de Estudios Biomédicos y Veterinarios, Instituto de Estudios Científicos y Tecnológicos, Universidad Nacional Experimental Simón Rodríguez, Caracas, Venezuela; ^2^Unidad de Toxoplasmosis y Protozoosis, Departamento de Parasitología, Centro Nacional de Microbiología, Instituto de Salud Carlos III, Calle Alejandro Casona 7, Piso 6D, 28035 Madrid, Spain; ^3^Sección de Inmunohistoquímica y Microscopía Electrónica, Instituto Anatomopatológico “José A. O'Daly”, Facultad de Medicina, Universidad Central de Venezuela, Caracas, Venezuela; ^4^Grupo de Inmunobiología, Centro de Estudios Biomédicos y Veterinarios, Instituto de Estudios Científicos y Tecnológicos, Universidad Nacional Experimental Simón Rodríguez, Caracas, Venezuela; ^5^Centro de Microscopía Electrónica “Mitsuo Ogura”, Facultad de Ciencias, Universidad Central de Venezuela, Caracas, Venezuela; ^6^Investigador Prometeo, Universidad de Las Fuerzas Armadas ESPE, Departamento de Ciencias de la Vida, Grupo de Investigación de Sanidad Animal y Humana (GISAH), Sangolquí, Ecuador

## Abstract

Trypanosomosis, a globally occurring parasitic disease, poses as a major obstacle to livestock production in tropical and subtropical regions resulting in tangible economic losses. In Latin America including Venezuela, trypanosomosis of ruminants is mainly caused by* Trypanosoma vivax*. Biologically active substances produced from trypanosomes, as well as host-trypanosome cellular interactions, contribute to the pathogenesis of anemia in an infection. The aim of this study was to examine with a scanning electron microscope the cellular interactions and alterations in ovine red blood cells (RBC) experimentally infected with* T. vivax*. Ovine infection resulted in changes of RBC shape as well as the formation of surface holes or vesicles. A frequent observation was the adhesion to the ovine RBC by the trypanosome's free flagellum, cell body, or attached flagellum in a process mediated by the filopodia emission from the trypanosome surface. The observed RBC alterations are caused by mechanical and biochemical damage from host-parasite interactions occurring in the bloodstream. The altered erythrocytes are prone to mononuclear phagocytic removal contributing to the hematocrit decrease during infection.

## 1. Introduction

In Venezuela, trypanosomosis, a globally occurring parasitic disease, in animals is mainly caused by* Trypanosoma vivax*. Trypanosomosis is responsible for tangible economic losses due to impaired livestock production. The detrimental effects produced by* T. vivax* in livestock include growth retardation, loss of body weight, low production of animal proteins (meat and milk) [1, 2], and diminished fertility [3]. These effects are responsible for increasing production costs through an increased need for treatment and veterinary care [4–6]. The economic implications of trypanosomosis in developing subtropical regions underscore the value in research covering the pathogenesis of* T. vivax*.

Animal trypanosomosis is characterized by periodic fevers, parasitemia, and anemia, changes in blood chemistry, body mass loss, and often death. The anemia developed by animals is considered the most characteristic symptom [7]. It seems that the anemia is hemolytic in nature, intravascular in the early stages of the disease, and extravascular in the chronic phase [6, 8].

Current experimental and clinical evidence on animal trypanosomosis suggests that the main pathogenetic mechanisms of anemia include hemolysis, red blood cells (RBC) sequestration, hemodilution, platelet aggregation, lipid peroxidation, bleeding, and bone marrow dysfunction [9, 10]. However, insufficient research has been done to associate the structural and biochemical changes in RBCs as consequences of the interaction between RBC and trypanosomes. For example, existing reports list the following as possible mechanisms responsible for RBC injury: mechanical damage produced by the interaction of trypanosomes with RBC [11], adhesion and membrane fusion mediated filopodia [12], immune complexes adsorbed to erythrocytes [13], and biologically active substances released by live or dead trypanosomes which produce erythrocyte injury [14–17].

In Venezuela,* T. vivax* is the causative agent of trypanosomosis in ruminants. The pathogenesis, characterized by anemia and fever, seriously compromises the animal's health [1, 6, 18]. The aim of this study was to examine, with a scanning electron microscope (SEM), the cellular interactions and alterations in ovine RBCs experimentally infected with a Venezuelan* T. vivax* isolate.

## 2. Material and Methods

### 2.1. Experimental Animals

A total of nine (9) crossbreed male sheep in reproductive age (30–35 kg body weight) were used in the study. Two of them were used as control and seven as the experimental group. Sheep in the experimental group were randomly selected and inoculated simultaneously with strain LIEM-176 of* T. vivax*. Both groups were housed in separate corrals covered with antimosquito nets to prevent contact with blood sucking flies and disease vectors. All the animals were fed concentrated food (Purina®, 500 g/animal/day), hay, and water* ad libitum*.

Both groups were clinically, parasitologically, and PCR examined one week before starting the study (preinfection period) in order to confirm the negativity to* T. vivax* and other hemoparasites. All tests were conducted at the Instituto de Estudios Científicos y Tecnológicos under veterinary supervision.

### 2.2. Trypanosomes

The strain LIEM-176 of* T. vivax* used in this work was previously morphometric and molecularly characterized [19–21], isolated from naturally infected cattle in the Trujillo State (Venezuela), and kindly provided by Drs. Glenda Moreno and Laura Moron of Universidad de Los Andes and expanded by inoculation into sheep.

### 2.3. Experimental Infection of Sheep

One mL of blood containing approximately 1 × 10^6^ blood trypomastigotes of* T. vivax* was inoculated intravenously in the jugular vein of each sheep of the experimental group. Control animals were inoculated with 1 mL of isotonic saline solution. Once inoculated, experimental and control groups were clinically and parasitologically examined every 4 days for 45 days. Values collected as indicators of* T. vivax* infection included the temperature, hematocrit, total plasma proteins, hemoglobin, and parasitemia by the Brener method [22].

During experimental infection, all animals were maintained under veterinary supervision to safeguard health and minimize animal suffering. Protocols used were approved by the Ethical Committee for Laboratory Animal Use under number 013-11 according to the Ethics Chart of animal experimentation.

### 2.4. Scanning Electron Microscopy (SEM)

Aliquots of blood from sheep experimentally infected with* T. vivax* taken every 4 days from the jugular vein (Vacutainer System®) and prefixed with Karnovsky fixative solution (2.5% glutaraldehyde and 4%* p*-formaldehyde in Millonig's phosphate buffer 0.10 M, pH 7.4) were contained in vacuum-glass tubes of 5 mL. The blood fixation process was done following the technique described by Boada-Sucre et al. [23]. Briefly, fixation began at the time of blood sampling for 1 hour at 4°C. Finally, 100 *μ*L of prefixed blood was spotted on to coverslips pretreated with poly-L-lysine 0.1% v/v in order to promote the adhesion of blood cells. The excess of glutaraldehyde was removed by washing with Millonig's phosphate buffer and the postfixation was performed with 1% m/v osmium tetroxide (OsO_4_) for 1 hour at 4°C. Osmium excess was removed by washing with deionized water and samples were dehydrated in increasing concentrations of ethanol at 4°C. The biological material adsorbed to the coverslips was dried by evaporation on a critical point dryer (Hitachi HCP-2) using carbon dioxide (CO_2_) as liquid transition fluid. Then, samples were mounted on the sample holder and covered with platinum ion (Electron Microscopy Science EMS-350). Observations and micrographic records were performed on a field emission scanning electron microscope (FE Hitachi S-4500) with an accelerating voltage of 5 keV.

### 2.5. Transmission Electron Microscopy (TEM)

The liver samples were processed for TEM using the standard methods described by Boada-Sucre et al., 1999 [23]. Pieces of liver were fixed at 2 mm in diameter with glutaraldehyde 2.5% and OsO_4_ 1% (Millonig-buffered solutions pH 7.4; 300 mOsm), dehydrated in increasing ethanol concentrations, and embedded in Epon 812. Sections were cut with a diamond knife in a RMC Ultramicrotome MT 7.000, and stained with uranyl acetate and lead citrate. Sections were observed with a Hitachi H-7100 transmission electron microscope (75 kV accelerating voltage).

### 2.6. Statistical Analysis

Each SEM preparation was examined 15, 30, or 45 days after infection. For each SEM preparation, one hundred trypomastigotes were counted and the proportion of observed parasite-RBC adhesion was quantified. For the adhered trypomastigotes, the method of adhesion was counted; the proportion of cell body adhesion, free flagellum adhesion, and attached flagellum adhesion was calculated. The proportions of* T. vivax* adhesion and the cell body structures involved in the attachment were compared using a two-tailed Fisher's Exact Test at a 95% confidence level. Differences between observations were considered statistically significant when *p* < 0.05.

## 3. Results

**Figure 1:**
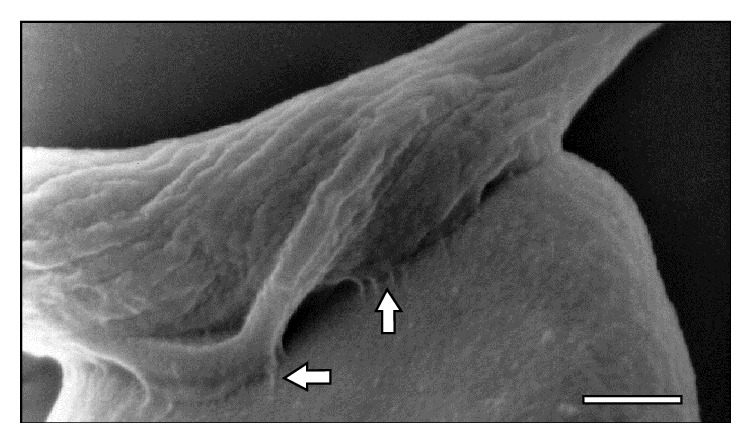
Scanning electron micrograph showing a large area (⇒) of* T. vivax* in close contact to sheep erythrocytes at 30 days after infection. Bar = 1.2 *μ*m.

**Figure 2 fig2:**
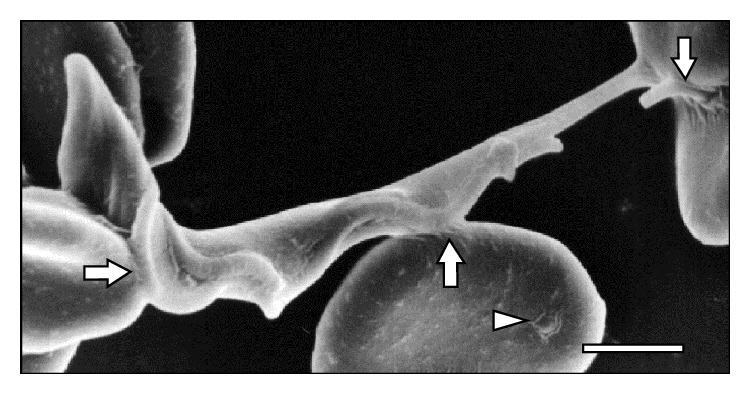
Adhesion of* T. vivax* to three RBCs through its body and flagellum (⇒). Note the presence of a vesicle (△) in one RBC at 15 days after infection. Bar = 1.4 *μ*m.

**Figure 3 fig3:**
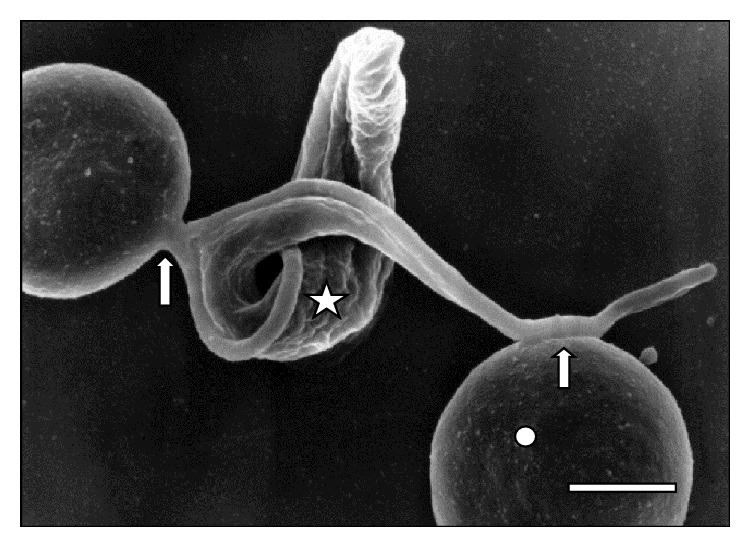
Attached (⇒) trypanosome (☆) to two sheep RBCs (◯) at 30 days of infection. Bar = 1.4 *μ*m.

**Figure 4 fig4:**
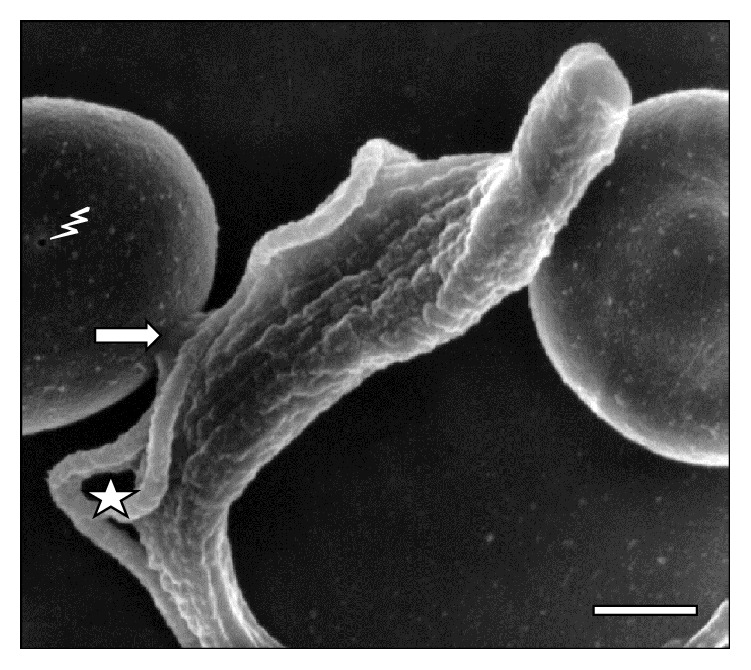
High resolution of a parasite in division process (☆) and contact (⇒) with an erythrocyte sheep at 45 days of infection. Note the holes in RBC (⚡). Bar = 2.0 *μ*m.

**Figure 5 fig5:**
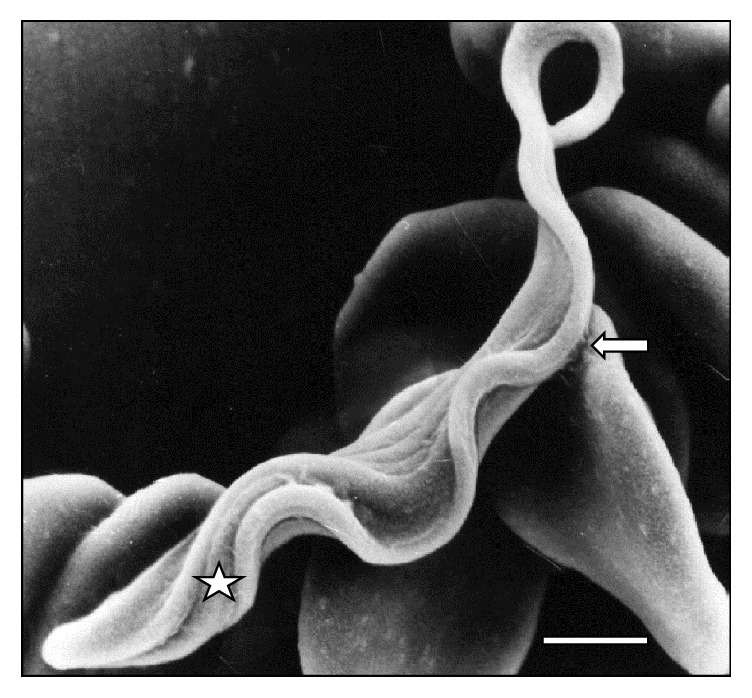
Scanning electron microscopy of adhesion (⇒) from one parasite in division (☆) to sheep erythrocyte at 45 days of infection. Bar = 2.0 *μ*m.

**Figure 6 fig6:**
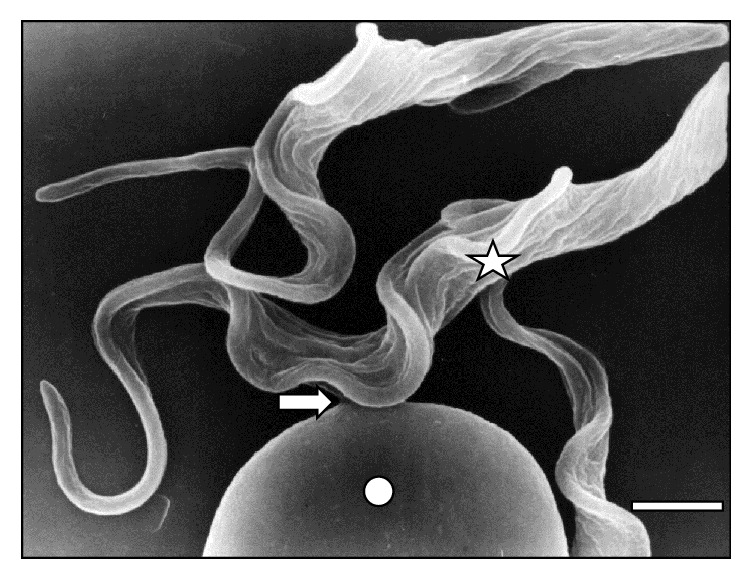
Electron microscopy of adhesion (⇒) of* T. vivax in division* (☆) to a sheep RBC (◯) through the attached flagellum at 30 days of infection. Bar = 2.0 *μ*m.

**Figure 7 fig7:**
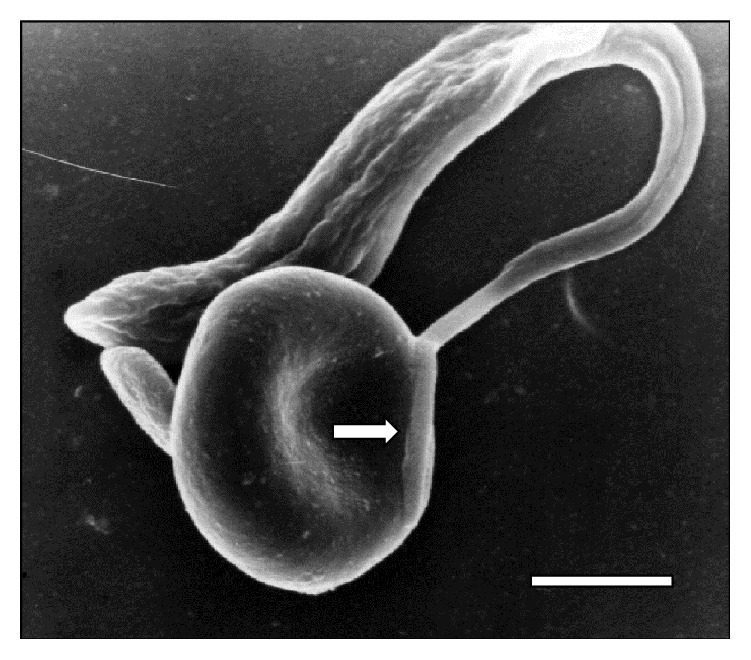
*T. vivax* flagellum contact (⇒) with a RBC at 15 days of inoculation. Bar = 1.7 *μ*m.

**Figure 8 fig8:**
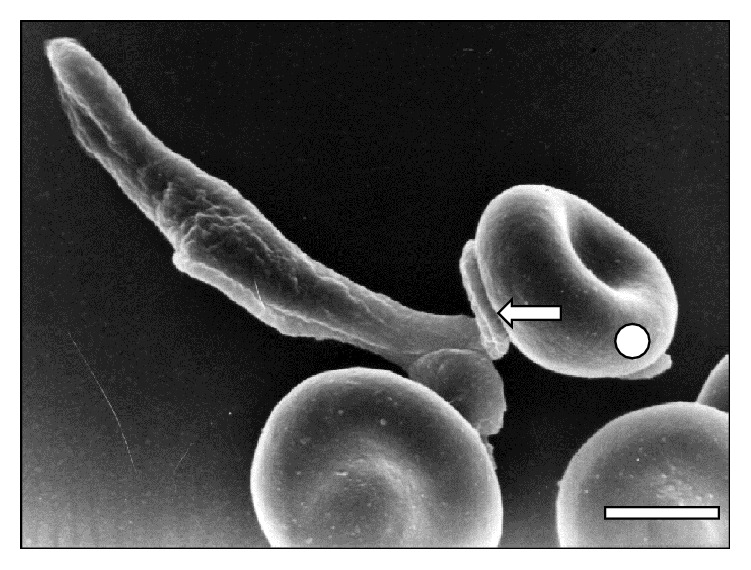
Scanning electron micrographs showing a large area of* T. vivax* flagellum in close adhesion (⇒) to a sheep erythrocyte (◯) at 30 days after infection. Bar = 1.6 *μ*m.

**Figure 9 fig9:**
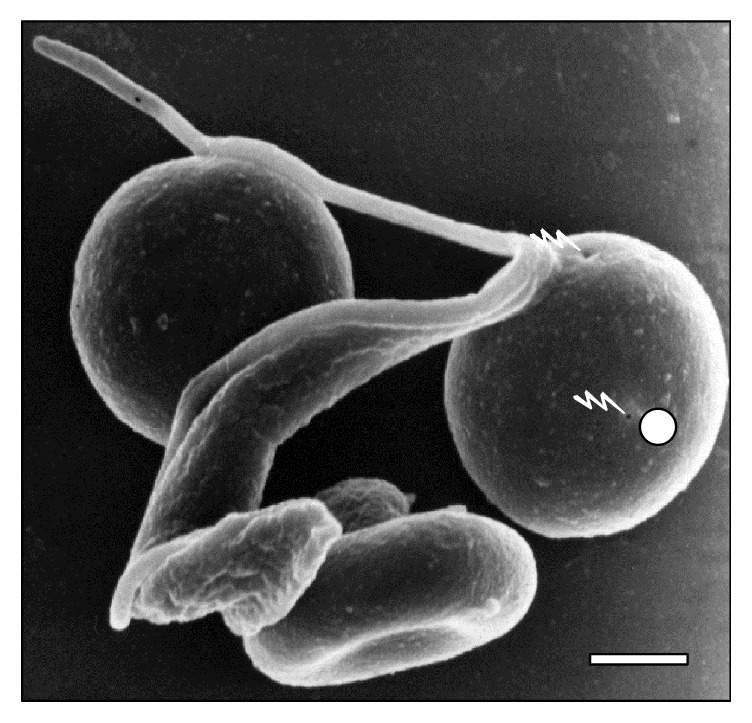
Scanning electron micrograph showing the adhesion of a* T. vivax* bloodstream trypomastigote to two sheep RBCs (◯) with holes (⚡) at 45 days of infection. The adhesion was through free flagellum and cell body. Bar = 2.0 *μ*m.

**Figure 10 fig10:**
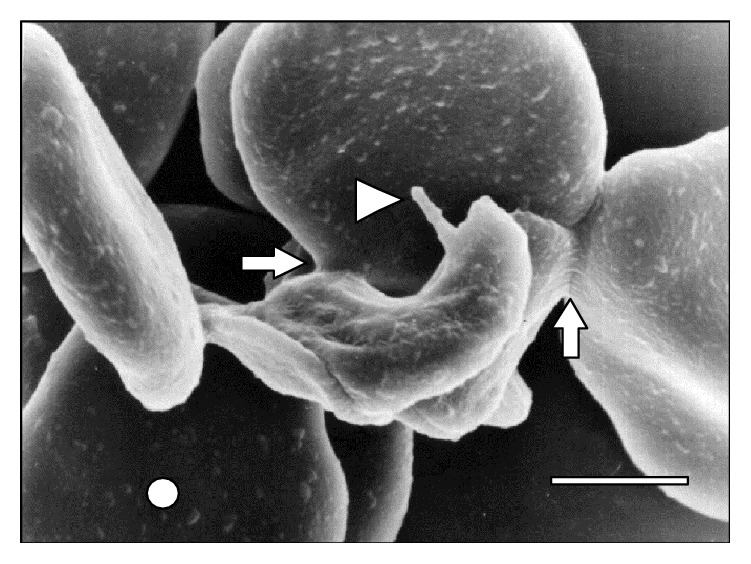
Trypomastigote in a close contact to a RBC cluster (◯) at 15 days after infection. Notice the emission of a pseudopod prolongation from* T. vivax* (△) and the adhesion of* T. vivax* trypomastigote to RBCs (⇒). Bar = 1.4 *μ*m.

**Figure 11:**
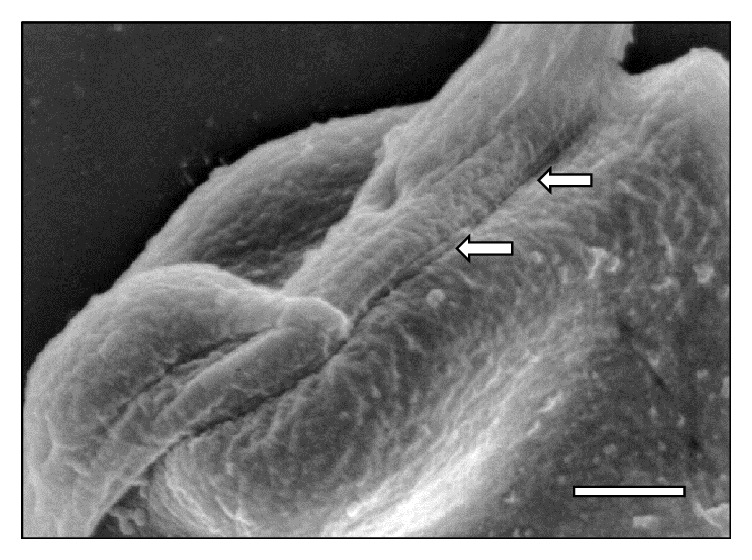
Scanning electron micrograph showing a large area (⇒) of* T. vivax* in close contact to an erythrocyte at 30 days after infection. Bar = 0.8 *μ*m.

Scanning electron microscopy showed the interaction of* T. vivax* with sheep RBCs. The interactions of* T. vivax* trypomastigotes with RBCs from experimentally infected sheep can be observed in the scanning electron micrographs of Figures 1 [Fig fig2] [Fig fig3] [Fig fig4] [Fig fig5] [Fig fig6] [Fig fig7] [Fig fig8] [Fig fig9] [Fig fig10]–11. As it can be observed, the interaction was achieved through the establishment of a very narrow cell contact and the subsequent development of RBC clusters. This adhesion was favored by the emission of filamentous and membranous projections through the trypanosome cell body (Figure 1). Figures 2 [Fig fig3] [Fig fig4] [Fig fig5]–6 show RBC adhesion mediated by the attached* T. vivax* flagellum. Free flagellum participation is observed in Figures 3 [Fig fig4] [Fig fig5] [Fig fig6] and 7 [Fig fig8]–9, while the direct involvement of* T. vivax* cell body is shown in Figures 1, 2 [Fig fig3] [Fig fig4] [Fig fig5] [Fig fig6] [Fig fig7] [Fig fig8], and 9 [Fig fig10]–11. This interaction between* T. vivax* and RBCs was measured as the proportion of* T. vivax* adhered at 15, 30, or 45 days after infection and is summarized in Table 1.

The proportion of adhered trypomastigotes at 15 days after infection is significantly higher than the proportion at 30 and 45 days after infection according to Fisher's Exact Test (*p* < 0.001). There was no significant difference between 30 and 45 days after infection (Table 1). Moreover, the parasite segments (cell body, free flagellum, and attached flagellum) adhered to the RBCs were quantified and the proportions were examined 15, 30, and 45 days after infection (Table 2).

Trypomastigotes adhered to sheep RBCs through the free flagellum at any time after infection are significantly higher (*p* < 0.0001) than the cell body and attached flagellum. Furthermore, the proportion of trypanosomes adhered to RBC through the attached flagellum is significantly lower (*p* < 0.0001) than the proportion of parasites adhered through the cell body.

The proportion of trypanosomes adhered to sheep RBC through the flagellum was very significant at day 15 when it was compared with the adhesion observed at days 30 and 45 (*p* < 0.0001). In addition, trypanosomes adhered through the cell body was significantly higher (*p* < 0.0001) at 15 and 45 days after infection, while the proportion of parasites adhered through the attached flagellum was significantly lower (*p* < 0.0001) at days 15 and 45 after infection.

**Figure 12:**
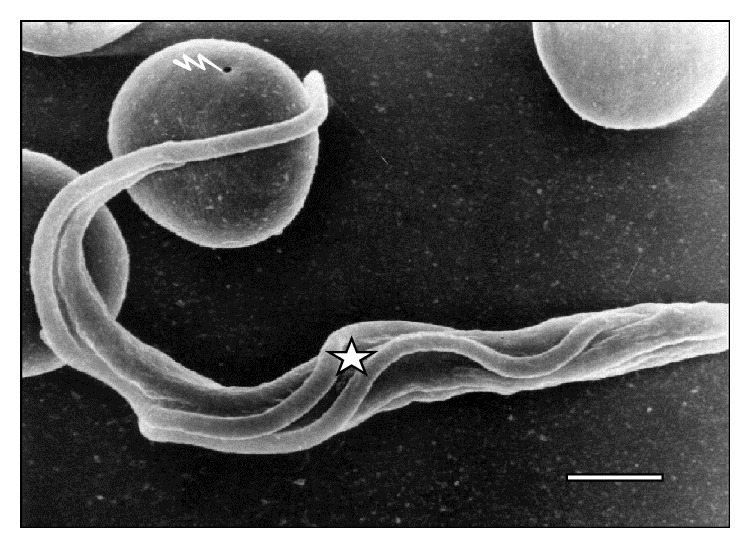
Scanning electron micrograph of a* T. vivax* trypomastigote in division process (☆) at 45 days of infection. Note the presence of holes on the RBC surface (⚡) and its round-morphology. Bar = 2.0 *μ*m.

**Figure 13:**
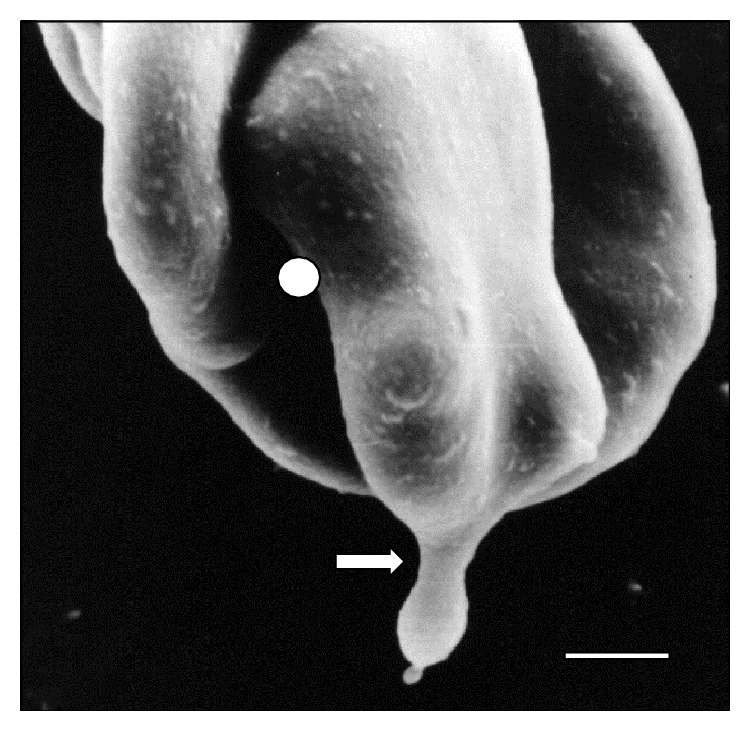
High resolution micrograph of a sheep RBC cluster showing a deformed RBC (◯) with small vesicles and emission of a pseudopod (⇒) at 15 days of infection. Bar = 1.3 *μ*m.

**Figure 14:**
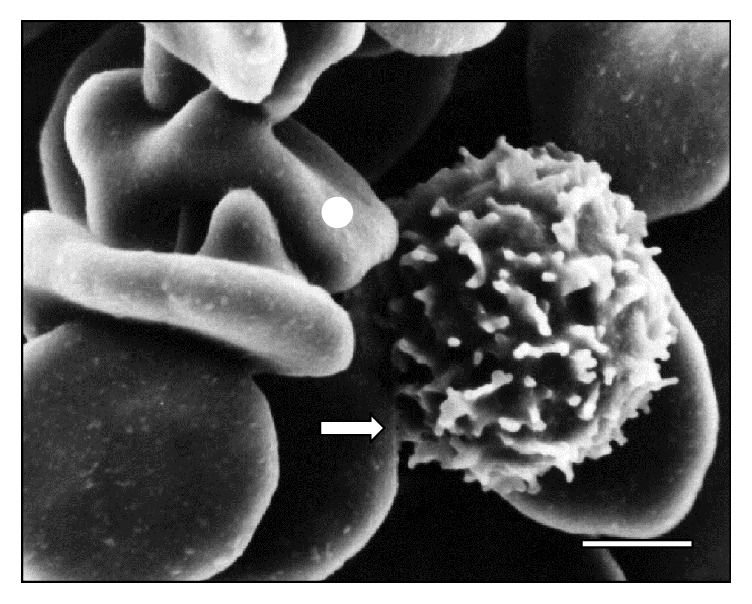
Adhesion (⇒) of one WBC to a deformed RBC (◯) in the peripheral blood of sheep at 15 days of infection with* T. vivax*. Bar = 1.0 *μ*m.

**Figure 15:**
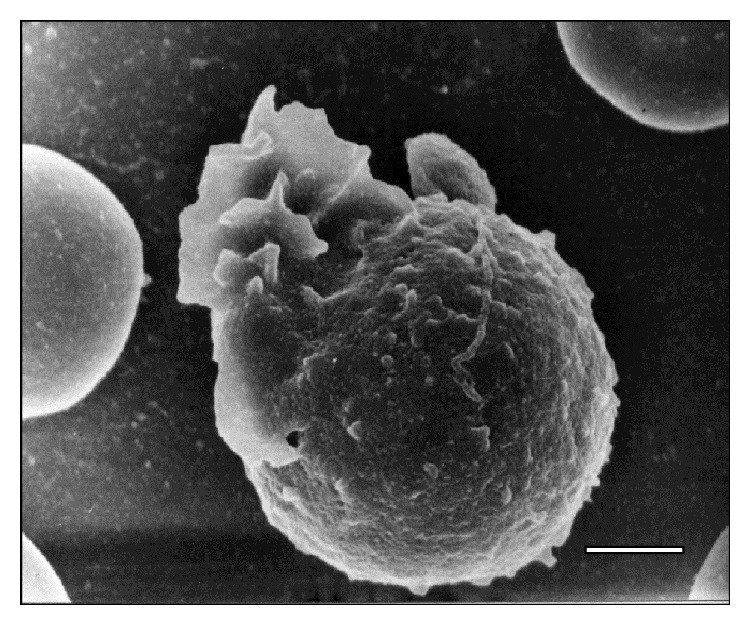
Electron micrograph showing an activated WBC in peripheral blood of a sheep at 15 days after infection with* T. vivax*. Bar = 2.5 *μ*m.

**Figure 16:**
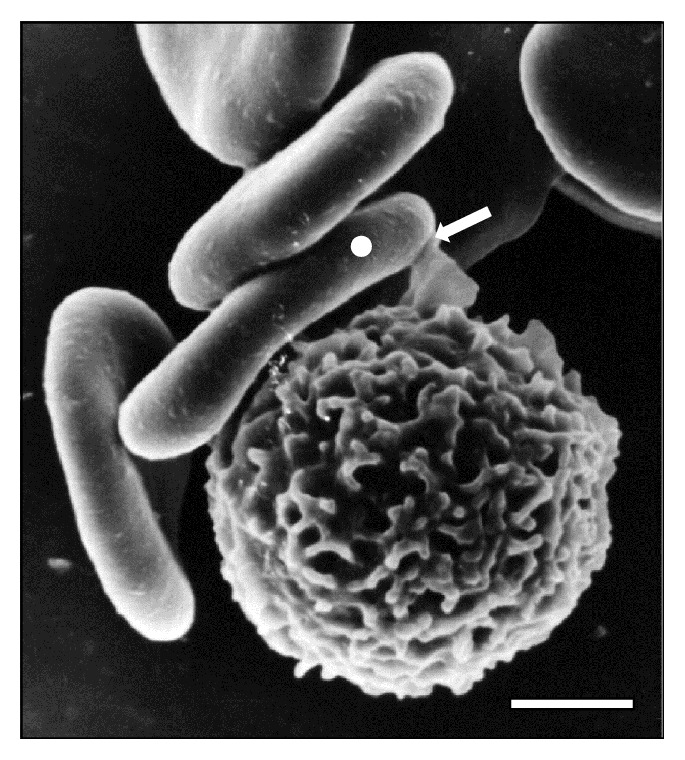
Adhesion (⇒) of WBC to RBC cluster (◯) in sheep peripheral blood at 15 days of infection with* T. vivax*. Bar = 2.0 *μ*m.

**Figure 17:**
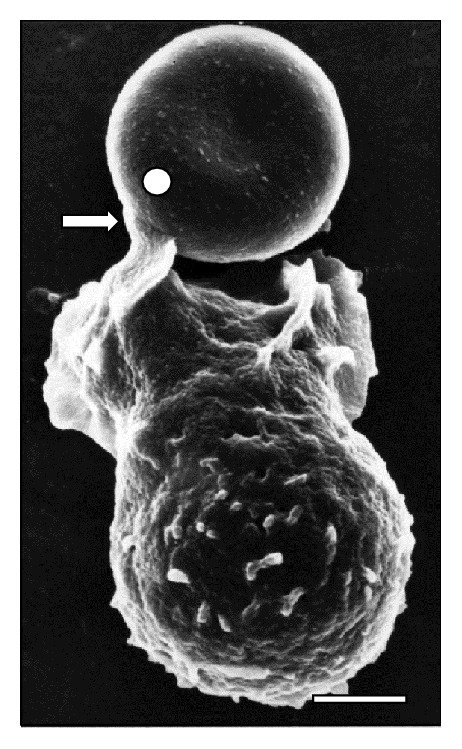
Phagocytosis (⇒) of a RBC (◯) by an activated WBC in peripheral blood of a sheep at 15 days of infection with* T. vivax.* Bar = 3.0 *μ*m.

**Figure 18:**
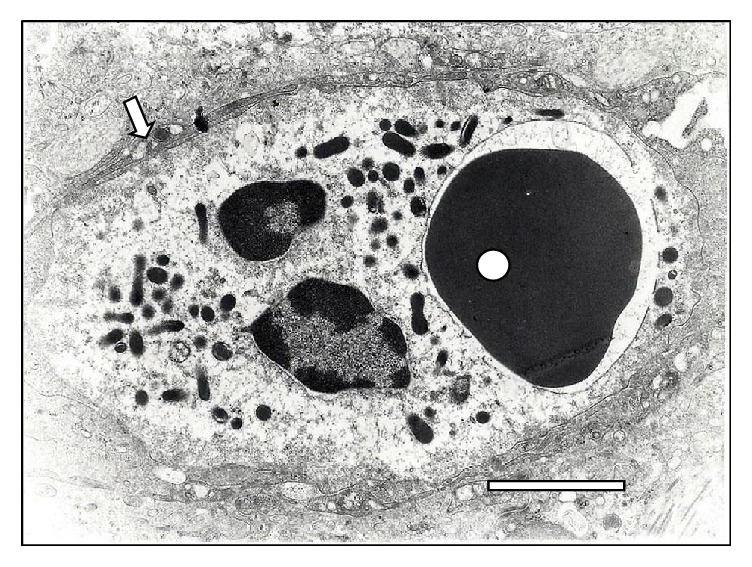
Transmission electron microscopy of a RBC phagocytosed (◯) by a polymorphonuclear cell-neutrophil (⇒) in the liver of a sheep infected with* T. vivax* at 15 days. Notice the RBC inside a phagocytic vacuole surrounded by lysosomes. Bar = 1.5 *μ*m.

Additionally, SEM images depict the emission of filopodia by* T. vivax* during the experimental infection process (Figure 10). The interaction of bloodstream trypanosomes with RBCs induces vesicle formation (Figure 2). Moreover, SEM images show pore formation on the erythrocyte surface during experimental infection (Figures 4, 9, and 12). Other common findings were morphological alterations in RBC (Figures 13 and 14), activated white blood cells (WBC) (Figure 15), WBC in contact with RBC (Figures 14 and 16), microspherocytosis (results not showed), and erythrophagocytosis process extravascularly (Figure 17) and intravascularly in liver sinusoid (Figure 18).

## 4. Discussion

In the RBC-*Trypanosoma* adhesion observed by SEM, morphological changes in the RBCs are found to be a contributing factor in the pathophysiology of anemia in sheep trypanosomosis by* T. vivax*.

In this paper, we have described the adhesion between* T. vivax* and sheep RBC mediated by the parasite's cell body, attached flagellum, and free flagellum. Free flagellum adhesion appears to be favored by the emission of filopodia and filamentous material of parasitic origin. Similar observations were reported between* T. brucei* and* T. evansi* to murine RBC [12, 13].

This adhesion of* T. vivax* to sheep RBC led to notable observations. First, Table 1 shows higher adhesion during the early stage of the disease compared to the latest stage, suggesting that the adhesion of* T. vivax* to RBC is inhibited by the sheep's antibodies against the parasite membrane. As it was described as a capping effect in* T. evansi*, mediated by antibodies [24].

Next, Table 2 shows higher adhesion of* T. vivax* to RBC through free flagellum than cell bodies or attached flagellum. This difference is remarkable since it may be due to the free flagellum's continuous whiplash movement.

The emission of filopodia by the African trypanosomes has been described by several researchers since the 1970s in infections caused by* T. b. brucei* and* T. b. rhodesiense* [25] and African isolates of* T. vivax* [26] and* T. venezuelense* (*T. evansi*) [27]. Furthermore, their involvement in the adhesion of* T. vivax* to the proboscide of Tsetse fly (*Glossina fuscipes*) has been documented [28]. The emission of these filamentous material and filopodia of parasitic origin has been described in phagocytes of cattle infected with* T. vivax* [29] and in the adhesion of* T. congolense* to endothelial cells from bovine aorta [30].

According to Rossi [13], filopodia and filamentous material help the trypanosome-RBC interaction in a process that is mediated by receptors on the erythrocyte surface. Additionally, the filamentous material could act by promoting the establishment of zip-type connections or hemidesmosome formations as it has been described in the adhesion of* T. congolense* to bovine endothelial cells [30]. Similarly,* T. vivax* is capable of developing this zip-type connection (Figure 12) and filopodia (Figure 3).

The close contact between trypanosomes and RBC via sialic acid receptors causes injuries to erythrocyte membranes at the point of contact [11]. In addition to the aforementioned RBC injuries, the apparent membrane fusion, changes in the oligosaccharide composition of the surface of RBC, and mechanical damage “pinching out” caused by the motility of the trypanosomes in the bloodstream [13, 17, 31] induce ultrastructural, biochemical, and antigenic alterations of the RBC, which increases erythrophagocytosis, as shown in Figure 16.

At the level of the RBC plasma membrane, the modification of its composition by trypanosomal antigens, such as neuraminidases [13], activates effector mechanisms of the immune system leading to the formation and adsorption of antigen-antibody complexes and complements on the surface [32]. This mechanism could explain the occurrence of holes in the RBC surface as is observed in Figures 15, 16, and 17. Those holes may facilitate erythrolysis and/or recognition and removal of RBC by mononuclear phagocytic system. A similar response to this erythrophagocytosis is the autoimmune mechanism observed in alterations of horse muscles naturally infected with* T. evansi* [33].

Furthermore, the action of biologically active substances secreted [34] or derived from live or dead trypanosomes [35–37], particularly sialidase secreted/excreted from* T. vivax,* in the bloodstream [18, 37, 38] contributes to the intravascular erythrophagocytosis [12, 38] described in this paper (Figures 17 and 18). In contrast, changes in the glycocalyx of RBCs induce extravascular erythrophagocytosis mediated by the mononuclear phagocyte system [10, 12, 39–41].

Sialidases secreted/excreted by trypanosomes have the ability to remove sialic acid residues from the RBC glycocalyx, enabling glycoconjugate degradation and elimination of fatty acids from cell membranes by other biologically active substances such as proteases, glycosidases, and phospholipases [15, 42, 43]. In this sense, Guegan et al. [18] characterized a multigenic family of transsialidases in* T. vivax* Y486, some of which are released and able to induce erythrophagocytosis by desialylating the glycophorins.

Cluster formations of thrombi composed of RBC and leukocytes (Figures 14 and 15) have also been described in infections caused by* T. brucei* and* T. evansi* and may complicate the pathophysiology of the ovine trypanosomosis caused by* T. vivax*. The systemic complications triggered by anemia together with microthrombi of parasitic origin not only compromise the normal gas and metabolites exchange between blood and host tissues by way of the occlusion of the vascular lumen of capillary, but also contribute to the cell necrosis aggravated by endothelial alterations that occur in the disease [12, 13, 41, 44, 45].

Forty-five years ago, Goodwin [31] pointed out that abnormalities of the RBC in trypanosomosis are a consequence of changes that occur in the small blood vessels. With the Venezuelan strain of* T. evansi*, this alteration in vessels was observed by Finol et al. [45]. Moreover, these authors concluded that the observed alterations on vessels contribute to modifications of the RBC morphological and cytoskeletal structure. The proteolytic lysosomal enzymes released into the circulation system from trypanosomes cleave sialic acid fractions [46] resulting in the vascular endothelium adopting a tortuous shape [47]. While observing the SEM images of sheep tissue experimentally infected with* T. vivax*, we found occurrences of the aforementioned endothelium damage in small vessels.

In experimental infection of mice with* T. evansi*, changes such as thickening of the basement membrane, proliferation, and vacuolization of endothelial cells and mechanical damage for action of trypanosome flagella and bodies also contribute in the modification of the erythrocyte cytoskeleton structure and morphology [13, 44, 47].

Furthermore, as it has been described in the infections of* Camelus dromedarius* [48] and rats with* T. evansi* [49], the infection of sheep with* T. vivax* could generate an oxidative stress with the generation of free radicals and superoxide reactive species that attack the proteins and lipid membranes with osmotic fragility. The peroxidation of lipids determines the degradation of polyunsaturated fatty acids with the interruption of the plasma membrane and a concomitant loss of its basic functions and features, as was described by a different author [15, 50, 51].

The RBC alterations described in this study, like emission of filopodia, vesicles, roughness, and holes on the erythrocyte surface are similar compared to those reported in* T. brucei* [12] and* T. evansi* infections [13].

## 5. Conclusion

The adhesion of* Trypanosoma vivax* (LIEM-176) to sheep erythrocytes described in this study is the first evidence for* T. vivax* infection. Moreover, the presence of holes and vesicles caused by the adhesion to RBC is an important factor to be considered in parasite-host interactions and contributes to anemia during trypanosomosis.

## Figures and Tables

**Table 1:** Percentage (%) of trypanosomes adhered to RBC or free, during infection process.

Days after infection	% of *T. vivax *adhered to RBC	% of *T. vivax *nonadhered to RBC
15	40	60
30	38	62
45	38	62

**Table 2:** Percentage (%) of trypanosomes adhered to RBC through cell body, flagellum, and attached flagellum during infection process.

Days after infection	Cell body	Free flagellum	Attached flagellum
15	12	20	8
30	11	18	9
45	12	18	8
